# Characterization and analysis of complete chloroplast genome of *Clausena anisum-olens* (Blanco) Merrill

**DOI:** 10.1080/23802359.2021.2009386

**Published:** 2021-12-20

**Authors:** Jin-jin Li, Chang Liu, Dan Wang, Dong-qin Guo, Hui-hui Du

**Affiliations:** a College of Biology and Food Engineering, Chongqing Three Gorges University, Chongqing, China; b Nanjing Institute for Comprehensive Utilization of Wild Plants, Nanjing, P. R. China

**Keywords:** *Clausena anisum-olens* (Blanco) Merrill, chloroplast genome, sequence, gene, phylogenetic analysis

## Abstract

In this study, the chloroplast genome (Cp) of *Clausena anisum-olens* ((Blanco) Merrill) was sequenced by high-throughput sequencing technology. We found that the length of the chloroplast genome of *Clausena anisum-olens* was 159,753 bp, and the total GC content was 38.7%, including a large single-copy (LSC) region of 87,623 bp, a small single-copy (SSC) region of 17,986 bp, and 27,072 bp pairs of reverse repeats (IRS). The Cp genome encoded 129 genes, containing 86 protein-coding, 37 tRNA, and 6 rRNA genes. Phylogenetic analysis of the genome sequence showed that *Clausena anisum-olens* was closely related to *Micromelum minutum* and *Glycosmis mauritiana.*

*Clausena anisum-olens* (Blanco) Merrill is an important *Clausena* genus in Rutaceae. The wild species are distributed in Taiwan of China and are cultivated in Guangdong, Guangxi, and Yunnan of China (Su et al. 2011), *Clausena anisum-olens* belongs to the same genus *Clausena* but has more wild resources than *Clausena lansium* (Su et al. 2011). *Clausena anisum-olens* fruit exerts phlegm and cough relief. The leaves and branches are used as a herbal medicine to relieve rheumatic pains (Wu 1995). Most of the studies on this species have been focused on their pharmacological activities and chemical composition analysis, but few involved genome analysis (Su and Liang 2010).

The chlorophyll in the leaves of *Clausena anisum-olens* has attracted considerable attention because of its beneficial pharmacological activities, such as abundant content of fat-soluble natural pigments, which have antiseptic, deodorization, antipyretic, and hemostatic effects. Herein, we report the identification and characterization of the complete chloroplast genome of *Clausena anisum-olens*, which provides valuable information on a large number of sequences. Our present findings will facilitate further investigations of the genome and the phylogenetic relationships of the Rutaceae family.

Fresh and healthy wampee leaves (104°65′68.07′′E, 23°12′99.61′′N) were collected from Wenshan, of Yunnan Province. The certified herbarium specimen (No. ZDQ17012) was collected and stored by Kunming Zhifen Biotechnology Co., Ltd. Total DNA was isolated from dried leaf materials according to a previously reported modified CTAB method (Yang et al. 2014) and sequenced by second-generation sequencing using Illumina HiSeq 2500 platform (Novogene, Tianjin, China). To decrease the redundant data, the original reads were filtered by Trimmomatic v.0.32 software with default parameters (Bolger et al. 2014). Then, the obtained clean reads were assembled into circular contigs using GetOrganelle (Jin et al. 2020) with *Clausena excavata* (No. NC_032685) as the reference. Finally, the cpDNA was annotated by the Dual Organellar GenoMe Annotator GeSeq (Tillich et al. 2017) and CpGAVAS2 (Shi et al. 2019). Finally, the annotated chloroplast genome was submitted to the GenBank (accession number: MZ460583).

The whole genome of *C. anisum-olens* has a typical tetragonal structure and a length of 159,753 bp. It consists of 87,623 bp large single-copy (LSC) region, 17,986 bp small single-copy (SSC) region and 27,072 bp reverse repeat regions (IRA and IRB). The total GC content was 37.8%. The CP genome was found to be composed of 129 genes, including 86 protein-coding, 37 tRNA, and 6 rRNA genes.

To determine the phylogenetic position of *C. anisum-olens*, 20 complete chloroplast genome sequences were downloaded from the NCBI database. The genome sequences were aligned with MAFFT version 7.427 (Katoh and Standley 2013), and then the maximum-likelihood (ML) tree was constructed by the RAxML (Stamatakis 2014) program with 1000 bootstrap replicates and the GTRGAMMAI model. Our results showed that *C. anisum-olens* was closely related to *Micromelum* and *Glycosmis* in the family of Rutaceae (Figure 1).

**Figure 1.**
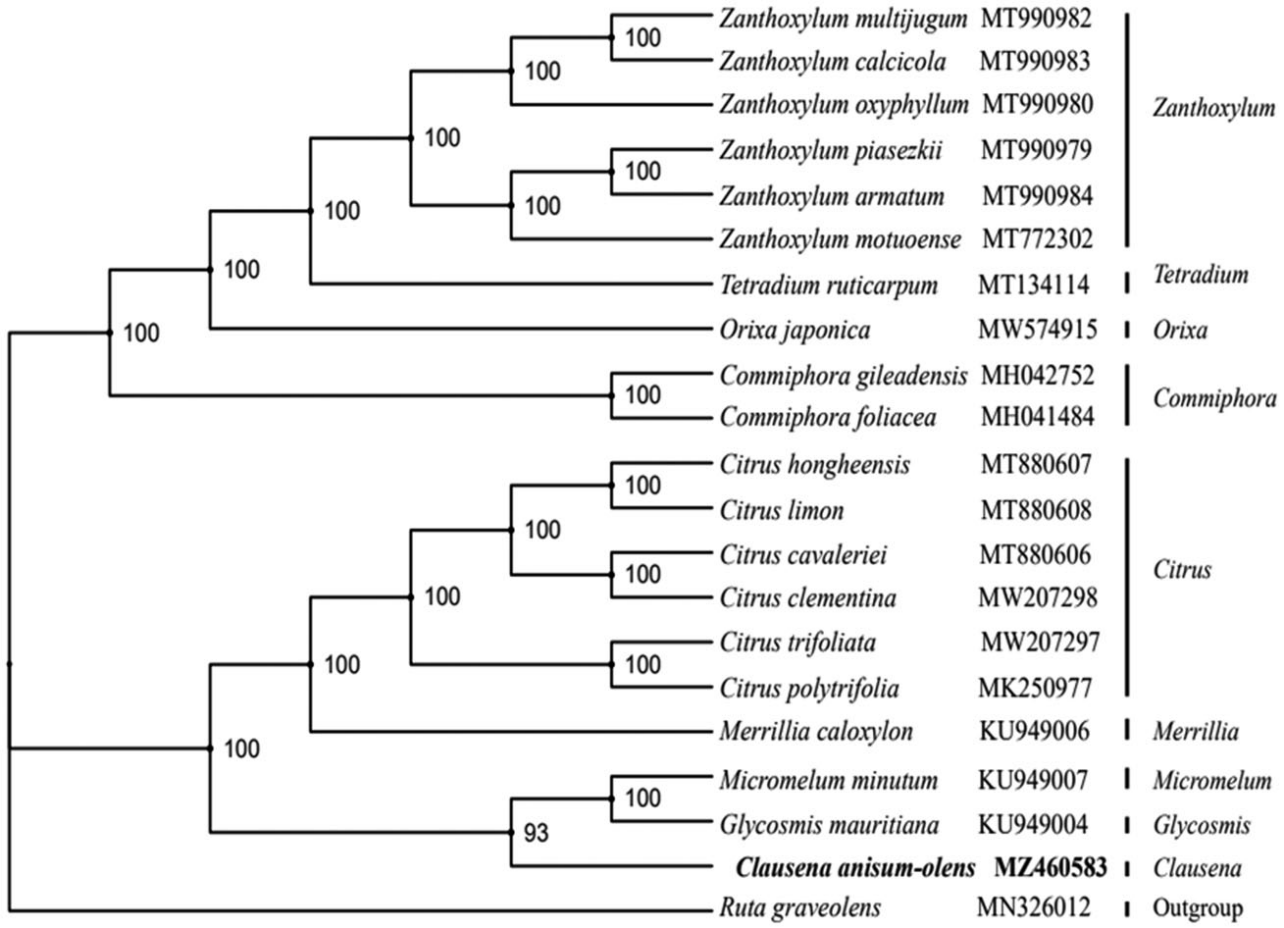
Phylogenetic tree reconstructed by maximum-likelihood (ML) analysis based on the complete chloroplast genome sequences of 21 species with *Ruta graveolens* as an outgroup. Bootstrap support values (1000 replicates) are displayed next to the nodes.

## Data Availability

The genome sequence data that support the findings of this study are openly available in the GenBank of NCBI at (https://www.ncbi.nlm.nih.gov/) under the accession no. MZ460583. The associated BioProject, SRA, and BioSample numbers are PRJNA758156, SRR15647457, and SAMN21013949, respectively.
